# Seed endophytic ammonia oxidizing bacteria in *Elymus nutans* transmit to offspring plants and contribute to nitrification in the root zone

**DOI:** 10.3389/fmicb.2022.1036897

**Published:** 2022-11-29

**Authors:** Danni Liang, Saman Bowatte

**Affiliations:** ^1^State Key Laboratory of Herbage Improvement and Grassland Agro-ecosystems, College of Pastoral Agriculture Science and Technology, Lanzhou University, Lanzhou, China; ^2^AgResearch Limited, Grasslands Research Centre, Palmerston North, New Zealand

**Keywords:** ammonia oxidizing bacteria, seed endophytes, nitrification, *Elymus nutans*, vertical transmission

## Abstract

**Background:**

Ammonia oxidizing bacteria (AOB) in soil are of great biological importance as they regulate the cycling of N in agroecosystems. Plants are known to harbor AOB but how they occupy the plant is an unresolved question.

**Methods:**

Metabarcoding studies were carried out using Illumina MiSeq sequencing to test the potential of seed vectored AOB exchange between plants and soil.

**Results and discussion:**

We found 27 sequences associated with AOB strains belonging to the genera *Nitrosospira, Nitrosovibrio*, and *Nitrosomonas* inhabiting *Elymus nutans* seeds collected from four geographically distanced alpine meadows. *Nitrosospira multiformis* was the most dominant across the four locations. The AOB community in *E. nutans* seeds was compared with that of the leaves, roots and soil in one location. Soil and seeds harbored a rich but dissimilar AOB community, and *Nitrosospira* sp. PJA1, *Nitrosospira* sp. Nsp17 and *Nitrosovibrio* sp. RY3C were present in all plant parts and soils. When *E. nutans* seeds were germinated in sterilized growth medium under greenhouse conditions, the AOB in seeds later appeared in leaves, roots and growth medium, and contributed to nitrification. Testing the AOB community of the second-generation seeds confirmed vertical transmission, but low richness was observed.

**Conclusion:**

These results suggest seed vectored AOB may play a critical role in N cycle.

## Introduction

Ammonia oxidizing bacteria (AOB) are of great biological importance as they regulate the cycling of nitrogen (N) in ecosystems. They are ubiquitous in soil (Fierer et al., 2009), freshwater (Li et al., 2018) and marine habitats (Shafiee et al., 2021), as well as wastewater treatment plants (Wang et al., 2012). AOB control the rate-limiting step in nitrification, by oxidizing ammonia to nitrite (NO_2_^–^). That NO_2_^–^ is then subsequently oxidized by bacterial NO_2_^–^ oxidizers to nitrate (NO_3_^–^). The NO_3_^–^-N produced by nitrification in soil can be taken up by plants, leached through soil profiles, or may provide a substrate for the production of nitrous oxide—a potent greenhouse gas. AOB activity is therefore directly linked to important ecosystem services such as the provision of N for plant growth and the control of N losses through leaching or gaseous emissions (Norton and Ouyang, 2019).

Ammonia oxidizing bacteria comprise several genera and strains in the phylum Proteobacteria, which can vary in the efficiency with which they facilitate nitrification (Prosser et al., 2014). Which strains are abundant and active, and therefore the rate and efficiency of nitrification, depends upon a number of factors that include the soil environment (Gannes et al., 2014), the identity of the plant growing in the soil (Ma et al., 2020), and the exudates it releases to the rhizosphere, including nitrification inhibitors (Subbarao et al., 2009). There is evidence that AOB also inhabit plant leaves (Papen et al., 2002; Bowatte et al., 2006, 2015) and roots (Briones et al., 2002; Bowatte et al., 2007), though it has yet to be explained how they came to occupy these plant parts. Transmission of soil and seed microbiota to seedlings has been reported by Rochefort et al. (2021); hence, the transmission of AOB to seedlings from soil, as well as from seeds, is possible. For example, several studies have shown that some seed bacteria are able to travel within the plant, exit the roots, and become functionally active in the rhizosphere. Puente et al. (2009) showed that bacteria transmitted from the seed to the rhizosphere of cardon cactus were able to mineralize the surrounding rock and improve nutrient absorption by the roots. White et al. (2018) reported that seeds vectored rhizobacteria, thereby facilitating organic N acquisition by grass seedlings—a phenomenon they later termed “rhizophagy.”

Our recent seed microbiome survey of *Elymus nutans* growing in alpine meadows (Guo et al., 2020) found operational taxonomic units (OTUs) closely matched to the *Nitrosomonadaceae* family, indicating AOB are inhabiting seeds. Here, we aim to confirm seed inhabitancy of AOB using Illumina sequencing of ammonia monooxygenase—a functional gene that encodes the first step of ammonia oxidation in AOB (Rotthauwe et al., 1997), and characterize their dynamics in *E. nutans* plants. *Elymus nutans* is the most dominant perennial forage grass that grows naturally in the alpine meadows of the Qinghai–Tibet Plateau (QTP), where we collected the seeds for the current study.

The N cycle is directly linked to the functional stability of ecosystems and many N-cycle processes are microbially mediated. Therefore, exploring the link between biogeochemical N-cycle processes and microbial community dynamics can provide a greater mechanistic understanding of the N cycle (Isobe and Ohte, 2014). Current understanding of N transformations is generally based on soil-dwelling microorganisms, but the importance of plant-dwelling microbial roles in the N cycle is emerging (Bowatte et al., 2015). This study examines the potential of seed-vectored AOB exchange between plants and soil. The findings will further advance our understanding of the fundamental concepts of biogeochemical N-cycle processes. We specifically addressed the following questions in this paper: (1) Is the presence of AOB in seeds of *E. nutans* geographically widespread? (2) Is there a core AOB community among the seeds, leaves, roots and root-zone soils of mature plants? (3) Are seed-borne AOB transmitted to offspring plants and their progeny? (4) Do seed-borne AOB contribute to nitrification?

## Materials and methods

### Ammonia oxidizing bacteria survey in seeds of *Elymus nutans*

The seeds of *E. nutans* were collected at ripening stage in September 2018 from four alpine meadows located on the QTP: Hongyuan (HY), Maqu (MQ), Luqu (LQ), and Guoluo (GL). The distances between sites are LQ-GL: 333 km, GL-MQ: 130 km, MQ-HY: 249 km, HY-LQ: 290 km. Details of the geography and climate of the four locations are provided in Supplementary Table 1. At each site, seeds were collected from three areas (10 × 10 m) approximately 80–100 m apart and treated as replicates. Each replicate sample contained seeds (approximately 300) collected from several (10–20) plant clumps growing within the same area. The seeds were transported in an icebox to the laboratory and stored at 4°C for later use. Seeds were surface sterilized as described in Guo et al. (2020) before the DNA extraction step.

### Ammonia oxidizing bacteria community in seeds, leaves, roots, and root-zone soil

Seeds, leaves, roots and root-zone soils of mature *E. nutans* plant clumps were collected to compare the AOB communities present in different plant parts and the soil. The samples were collected from an alpine meadow at the Azi research station in Maqu county (MQ—Supplementary Table 1) on the eastern QTP in September 2019. Three mature *E. nutans* plant clumps at the site were sampled. The plant clumps were approximately 80–100 m apart. The whole plant clump was removed with attached soils to a 10 cm soil depth using a 5 cm diameter soil corer. Samples were transported in an icebox to the laboratory, and then the seeds and leaves were separated. Roots of the *E. nutans* plants were carefully separated by holding the above ground parts of the plant clump. Soils attached to the roots were taken as the root-zone soil sample. Roots were washed with sterilized distilled water. Seeds, roots and leaves were then surface-sterilized (Guo et al., 2020). All samples were stored at -20°C until further use.

### Seed-borne ammonia oxidizing bacteria transmission to offspring plants

The experiment was carried out in a ventilated greenhouse at the Yuzhong campus of Lanzhou University, Lanzhou, China, from May to July 2020. The greenhouse was maintained at a light intensity of 800 μmol m^–2^ s^–1^, a temperature regime of 16°C at night and 25°C during the day, and a 16-h photoperiod. The *E. nutans* seeds collected at the Azi research station in 2019 were used in this study. We used sterilized coir (heated at 150°C for 48 h) as the plant growth medium. Absence of AOB in coir was confirmed by *amoA* gene targeted PCR analysis prior to seed sowing, and later by Illumina sequencing analysis (see details below). First, 600 g sterilized coir was filled into rectangular plastic plant pots (34 × 19 × 11 cm length, width and height, respectively) that were sterilized by wiping with 75% ethanol. The surface-sterilized *E. nutans* seeds were germinated on sterile moist filter paper with sterilized water, and after 5 days of germination, 10 seedlings were transplanted into the sterilized coir growth medium. Three replicate pots were maintained, watered once a week with sterilized distilled water, and sterilized Hogland nutrient solution (Beijing Solarbio Science & Technology Co., Beijing, China) was added every 2 weeks. A coir sample at the beginning, and seedlings at 12 and 39 days after transplanting, were removed for AOB testing. The leaves were cut, roots were separated from the coir, and the coir attached to roots was considered as the root medium. Surface-sterilized roots and leaves (as described earlier) and the coir sample were stored at -20°C for further use. Three separate pots with sterilized coir without seedlings were maintained throughout the study as the control treatment. After sample collection at 39 days, the plants were maintained in the greenhouse for a further 15 months for second-generation seed collection.

### DNA extraction

Approximately 0.5 g seeds, leaves, roots, soil and coir samples were used for total genomic DNA extraction by the DNeasy Power Soil Kit (QIAGEN, Inc., Netherlands). Following the manufacturer’s instructions, the extracted DNA was dissolved in 100 μL of the DNA elution solution. Preliminary experiments carried out at Shanghai Personal Biotechnology Co., Ltd., found the DNeasy Power Soil Kit to be best compared to commercial plant DNA extraction kits for DNA extraction for microbial analysis from seed, leaves and roots (personal communication, Shanghai Personal Biotechnology Co., Ltd., Shanghai, China). The quantity and quality of extracted DNA were measured using a NanoDrop ND-1000 spectrophotometer (Thermo Fisher Scientific, Waltham, MA, USA) and agarose gel electrophoresis, respectively. DNA samples were stored at -20°C prior to further analysis.

### Illumina MiSeq sequencing of ammonia oxidizing bacteria

PCR amplification of ammonia oxidizing bacterial *amoA* genes was performed with primers *amoA*-1F (5′-GGGGTTTCTACTGGTGGT-3′) and *amoA*-2R (5′-CCCCTCKGSAAAGCCTTCTTC-3′) (Rotthauwe et al., 1997). Sample-specific 7-bp barcodes were incorporated into the primers for multiplex sequencing. Thermal cycling consisted of initial denaturation at 98°C for 5 min, followed by 25 cycles consisting of denaturation at 98°C for 30 s, annealing at 53°C for 30 s, and extension at 72°C for 45 s, with a final extension of 5 min at 72°C. PCR amplicons were purified with Vazyme VAHTSTM DNA Clean Beads (Vazyme, Nanjing, China) and quantified using the Quant-iT PicoGreen dsDNA Assay Kit (Invitrogen, Carlsbad, CA, USA). After the individual quantification step, amplicons were pooled in equal amounts, and pair-end 2 × 300 bp sequencing was performed using the Illumina MiSeq platform with the MiSeq Reagent Kit v3 at Shanghai Personal Biotechnology Co., Ltd (Shanghai, China). Sequencing reads were assigned to each sample by a certain barcode and then processed in QIIME 2 software (Bolyen et al., 2018). The Vsearch tool was used to remove the low-quality reads (quality score < 20, length < 150 bp) and chimeras. The sequences were joined prior to taxonomy assignment, the length of high-quality sequences for classification was 491 bp. The remaining high-quality sequences were assigned to OTUs at a 97% similarity level using the UPARSE pipeline (Edgar, 2013).

Accession numbers: the AOB sequences retrieved in this study have been deposited in NCBI GenBank under the SRA accession number PRJNA632329 for AOB in *E. nutans* seeds across the four locations, PRJNA796752 for different parts of *E. nutans* at site MQ, and PRJNA796717 for AOB in *E. nutans* seedlings at day 12 and day 39.

### Phylogenetic analysis

Phylogenetic affiliation of the sequences associated with AOB strains identified in the seeds of *E. nutans* growing in the four locations, and the AOB inhabiting different plant parts of *E. nutans*, were examined by constructing a neighbor-joining tree using the partial *amoA* sequences. The reference *amoA* sequences were obtained from Boyle-Yarwood et al. (2008) and Chen et al. (2011). The tree topology was checked by the neighbor-joining algorithm and the minimum-evolution method (Tamura et al., 2004), which was conducted in MEGA version 7.0 (Kumar et al., 2016) by performing 1,000 bootstrap replicates.

### Diversity analysis

The shared and unique AOBs in seed, leaf, root and soil were identified via a Venn diagram by Origin 2021 software. The diversity analysis was carried out using 9,997 reads per sample (see rarefaction curves in Supplementary Figure 1). The alpha diversity of the AOB communities in seed, leaf, root and soil were examined by estimating the Chao 1, Shannon and Simpson diversity using QIIME 2 software (Chao, 1984). The significance of the differences of relative abundances of sequences associated with AOB observed in four geographic locations were statistically tested by the Kruskal-Wallis rank sum test and Dunnett’s test. The beta diversity of the AOB communities in seed, leaf, root and soil AOB communities were assessed by non-metric multidimensional scaling (NMDS) using a weighted uniFrac distance matrix using isoMDS function in MASS package in R software (R Core Team, 2020), the differences of AOB communities between different plant parts of *Elymus nutans* were statistically tested by ANOSIM (analysis of similarities) with 999 permutations using QIIME 2 software.

### Contribution of seed-borne ammonia oxidizing bacteria to nitrification in the rhizosphere

A second pot experiment similar to that described above was carried out in August 2021 to test whether the AOB that migrated from seeds to the root zone of the seedlings contribute to nitrification. Fifty surface-sterilized *E. nutans* seeds collected from the Azi research station in 2019 (the AOB community in the seeds had already been characterized) were used in this experiment. Ten seeds were grown in sterilized coir medium in a pot with same dimensions as described above. There were five replicate pots with and without seedlings (control). The pots were watered once a week with sterilized distilled water, and sterilized Hoagland nutrient solution without N (Beijing Solarbio Science & Technology Co., Beijing, China) was added every 2 weeks. At the start of the experiment, 100 mL of 1 M (NH_4_)_2_SO_4_ was added to all the pots. The NO_2_^–^ and NO_3_^–^ formation from ammonium (NH_4_^+^) added to the coir was monitored using ion exchange membrane (IEM) probes as described by Bowatte et al. (2008). The IEM-adsorbed NO_2_^–^ and NO_3_^–^ were extracted with 2 M KCl. The NO_2_^–^ and NO_3_^–^ contents in the extracts were measured colorimetrically as described by Wang et al. (2002) and O’Sullivan et al. (2017), respectively.

## Results

### Ammonia oxidizing bacteria survey in seeds of *Elymus nutans*

**FIGURE 1:**
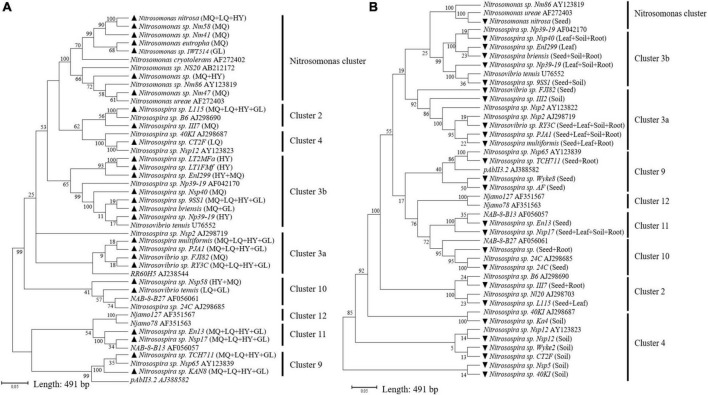
Neighbor-joining tree constructed with OTUs consisting of 491 bp fragment of the *amoA* sequences retrieved from *E. nutans* seeds at four locations **(A)** and different plant parts of *E. nutans* growing in alpine meadows at Maqu Azi station **(B)**. Filled black triangles in panel **(A)** represent AOB species in *E. nutans* seeds at the four locations and the capital letters in parentheses represent different sampling sites (MQ = Maqu; LQ = Luqu; HY = Hongyuan; GL = Guoluo). Filled black inverted triangles in panel **(B)** represent AOB species from different plant parts of *E. nutans* at Maqu Azi station. The reference sequences for AOB clusters are from Boyle-Yarwood et al. (2008) and Chen et al. (2011).

Illumina MiSeq sequencing resulted in a total of 276,569 high quality *amoA* sequencing reads from 12 samples across all four locations, with an average of 23,047 reads per sample. These sequences were clustered to 40,492 AOB OTUs that were classified into 27 sequences associated with AOB strains belonging to three genera: *Nitrosospira, Nitrosovibrio* and *Nitrosomonas* (Figure 1A and Supplementary Table 2). The sequencing revealed that *E. nutans* seeds collected from all four locations harbored AOB. The highest diversity of seed AOB was observed at MQ, where 21 sequences associated with AOB strains were observed, whereas 11, 12, and 13 sequences associated with AOB strains were present at LQ, GL, and HY, respectively (Figure 1A and Supplementary Table 2). Across all sampling sites, *Nitrosospira multiformis, Nitrosospira* sp. L115 and *Nitrosospira* sp. TCH711 were the most abundant AOB inside the seeds, with average relative abundances of 58.5 ± 8.3%, 18.6 ± 3.9%, and 7.2 ± 0.8%, respectively (Supplementary Table 2). The phylogenetic tree identified that most AOB of *E. nutans* seeds belonged to *Nitrosospira* cluster 3b (Figure 1A). The remaining AOB of *E. nutans* seeds were grouped within *Nitrosospira* clusters 3a, 10, 11, and 1, and *Nitrosomonas* cluster. Among the 27 different AOBs, *Nitrosospira* sp. L115, *Nitrosospira multiformis, Nitrosospira* sp. Nsp17, *Nitrosospir*a sp. TCH711, *Nitrosospira* sp. KAN8, *Nitrosospira* sp. PJA1 and *Nitrosovibrio* sp. RY3C were found in seeds collected from all four locations.

### Ammonia oxidizing bacteria communities in seeds, leaves, roots, and root-zone soil

**FIGURE 2:**
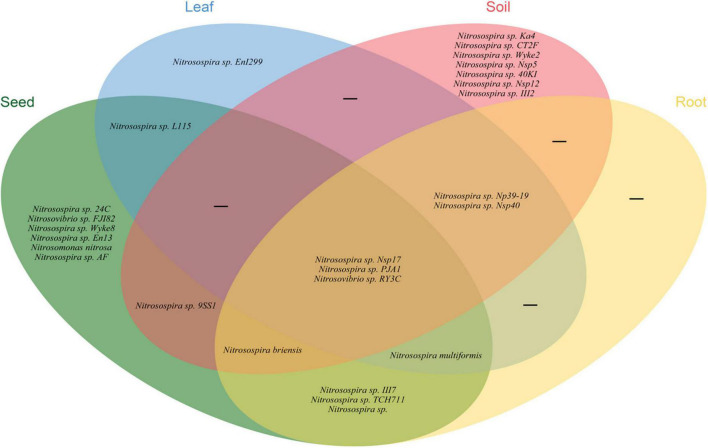
Venn diagram illustrating the unique, shared and core ammonia oxidizing bacteria in different plant parts and soils of *E. nutans* growing at Maqu Azi station on the QTP.

**FIGURE 3:**
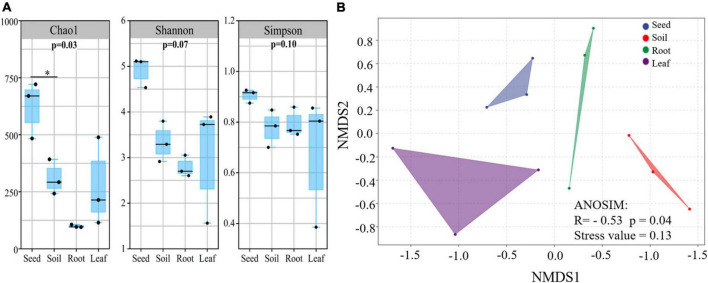
The alpha **(A)** and beta **(B)** diversity of ammonia oxidizing bacteria in different plant parts and soil of *E. nutans* growing at Maqu Azi station on the QTP (*n* = 3). Alpha diversity indices: Chao 1, Shannon and Simpson diversity were estimated using QIIME 2 software. Beta diversity was assessed by non-metric multidimensional scaling (NMDS) using a weighted uniFrac distance matrix using isoMDS function in MASS package in R software.

Illumina MiSeq sequencing resulted in a total of 534,263 high-quality *amoA* sequencing reads across all 12 samples (three replicates of leaf, seed, roots and soil), with an average of 44,521 reads per sample. These sequences were clustered to 4,647 AOB OTUs that were classified into 26 sequences associated with AOB strains belonging to three genera: *Nitrosospira, Nitrosovibrio*, and *Nitrosomonas* (Figure 1B and Supplementary Table 3). The *amoA* sequencing confirmed that *E. nutans* plants harbor AOB in seeds, leaves, roots and root-zone soil. There were more AOB strain types found inside seeds (16) than in leaves (8), roots (10), and root-zone soil (14). There were AOB common to seeds, leaves, roots, and root-zone soil (Figure 2) —namely, *Nitrosospira* sp. PJA1, *Nitrosospira* sp. Nsp17, and *Nitrosovibrio* sp. RY3C. The Chao1, Shannon and Simpson diversity indices indicated that the α-diversity of the seed AOB community was higher than the AOB communities in leaf, root and soil (Figure 3A). The β-diversity examined by the NMDS analysis indicated distinct AOB communities in seeds, leaf, root and soil (Figure 3B).

### Seed-borne ammonia oxidizing bacteria transmission to offspring plants and seeds

Illumina MiSeq sequencing resulted in a total of 649,687 high-quality *amoA* sequencing reads across all 18 samples (coir, 12 and 39 day-old plants), with an average 36,093 reads per sample. These sequences were clustered to 4,268 AOB OTUs that were classified into 31 sequences associated with AOB strains belonging to three genera: *Nitrosospira, Nitrosovibrio* and *Nitrosomonas* (Table 1 and Supplementary Table 4). The *amoA* sequence reads were not observed from 12-day plant leaf DNA samples, but there were 22 AOB observed from roots. All 11 AOB species found in root medium in 12-day plants were also found in roots. There were seven AOB identified from leaf DNA samples from 39-day plants. There were six and seven AOB species identified from roots and root medium DNA samples from 39-day plants (Table 1 and Supplementary Table 4).

**TABLE 1:** The transmission of ammonia oxidizing bacteria in *Elymus nutans* seeds to leaf, roots, root medium of offspring plants at 12 and 39 days and progeny.

Ammonia oxidizing bacteria	Day 12			Day 39			15 months
	Leaf	Root	Root medium	Leaf	Root	Root medium	Second generation seeds
*Nitrosomonas nitrosa*	–	+	+	–	–	–	+
*Nitrosomonas* sp. *LT-4*	–	+	–	–	–	–	–
*Nitrosospira briensis*	–	+	–	–	–	–	+
*Nitrosospira multiformis*	–	+	+	+	–	+	–
*Nitrosospira* sp.	–	+	+	–	–	–	–
*Nitrosospira* sp. *24C*	–	–	–	–	–	–	–
*Nitrosospira* sp. *40KI*	–	+	–	–	–	–	–
*Nitrosospira* sp. *9SS1*	–	–	–	–	–	–	–
*Nitrosospira* sp. *AF*	–	–	–	–	–	–	–
*Nitrosospira* sp. *B6*	–	–	–	+	–	–	–
*Nitrosospira* sp. *CT2F*	–	+	–	–	–	–	–
*Nitrosospira* sp. *En13*	–	–	–	–	–	–	–
*Nitrosospira* sp. *EnI299*	–	–	–	–	+	–	–
*Nitrosospira* sp. *III2*	–	+	+	–	–	–	–
*Nitrosospira* sp. *III7*	–	+	+	+	–	–	–
*Nitrosospira* sp. *Ka3*	–	+	–	–	–	–	–
*Nitrosospira* sp. *Ka4*	–	+	–	–	–	–	–
*Nitrosospira* sp. *L115*	–	–	–	–	–	–	–
*Nitrosospira* sp. *Nl5*	–	–	–	–	–	+	–
*Nitrosospira* sp. *Np39-19*	–	+	–	+	+	+	–
*Nitrosospira* sp. *Nsp12*	–	+	+	–	–	–	–
*Nitrosospira* sp. *Nsp17*	–	+	+	+	+	+	+
*Nitrosospira* sp. *Nsp40*	–	+	–	–	+	+	–
*Nitrosospira* sp. *Nsp5*	–	+	–	–	–	–	–
*Nitrosospira* sp. *Nsp57*	–	+	+	–	–	–	–
*Nitrosospira* sp. *PJA1*	–	+	+	+	+	+	–
*Nitrosospira* sp. *TCH711*	–	+	+	–	–	–	–
*Nitrosospira* sp. *Wyke2*	–	+	+	–	–	–	–
*Nitrosospira* sp. *Wyke8*	–	+	–	–	–	–	–
*Nitrosovibrio* sp. *FJI82*	–	–	–	–	–	–	–
*Nitrosovibrio* sp. *RY3C*	–	+	–	+	+	+	–

The + or − symbols indicate the presence or absence, respectively, of specific AOB in samples.

Illumina MiSeq sequencing resulted in a total of 143,916 high-quality *amoA* sequencing reads from second-generation seed DNA samples. These sequences were clustered to 359 AOB OTUs that were classified into three sequences associated with AOB strains: *Nitrosospira briensis, Nitrosomonas nitrosa* and *Nitrosospira* sp. Nsp17 (Supplementary Table 5).

### Contribution of seed-borne ammonia oxidizing bacteria to nitrification in the rhizosphere

**FIGURE 4:**
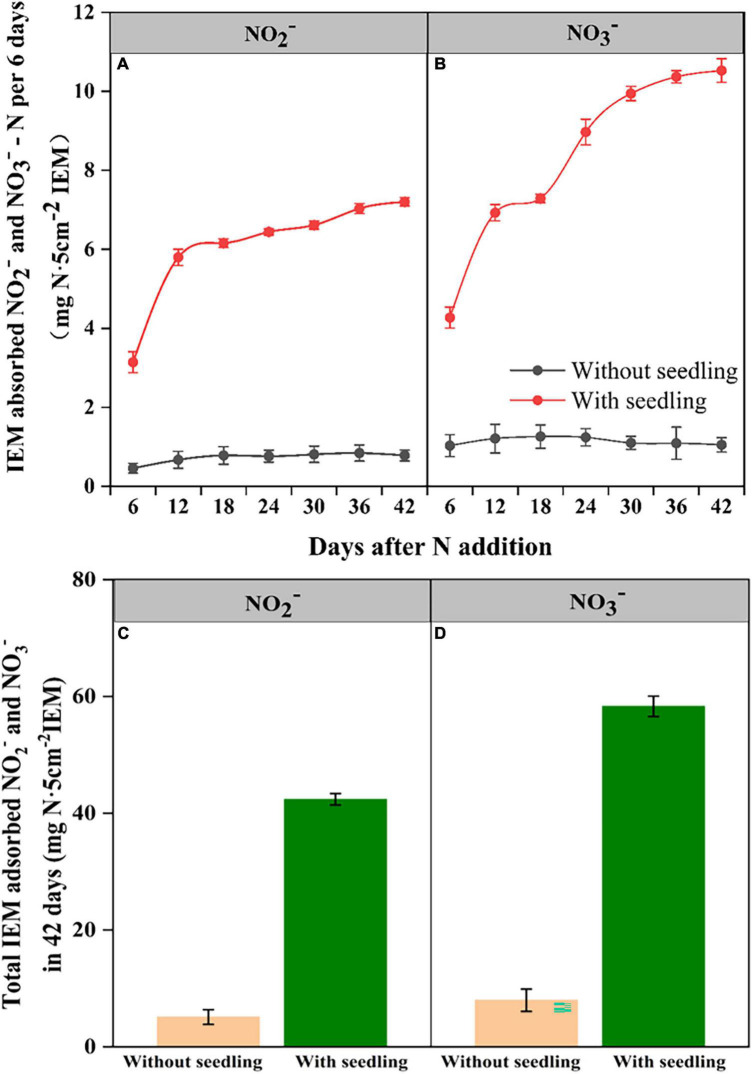
Ion exchange membrane (IEM) adsorbed NO_2_^–^-N **(A)** and NO_3_^–^-N **(B)** from the growth medium of *E. nutans* seedlings growing over 42 days and the cumulative NO_2_^–^-N **(C)** and NO_3_^–^-N **(D)** adsorbed over 42 days (*n* = 5).

Ion exchange resin membranes buried in coir medium adsorbed NO_2_^–^ and NO_3_^–^ from both control and seedling pots (Figure 4). The IEM-adsorbed N levels remained similarly low in control pots during the experimental period, suggesting trivial levels of NO_2_^–^-N and NO_3_^–^-N were constituted in the coir medium. In contrast, in pots with seedlings, IEM-adsorbed NO_2_^–^ and NO_3_^–^ levels increased as the seedling grew, indicating nitrification was occurring. As the medium was sterile, the increase in NO_2_^–^ and NO_3_^–^ in seedling pots should be due to the nitrifiers that have migrated to the coir medium from seedlings carrying out the ammonia and NO_2_^–^ oxidation.

## Discussion

Ammonia oxidizing bacteria are ubiquitous in the environment and their habitation in plants has also been reported. However, how the harboring of AOB by plants comes about is an unresolved question. Our results for the first time provide empirical evidence for the ability of plants to introduce AOB to offspring plants as well as soil via seeds. It is well established that various types of bacteria inhabit seeds and that these can later appear in the host plant (Simonin et al., 2022). However, the novel finding presented here is that seeds host bacteria that influence soil N cycling where these then become active and influential in the rhizosphere. Our previous study on a seed microbiome survey using 16S RNA gene sequencing (Guo et al., 2020) identified OTUs closely associated with *Nitrosomonadaceae* family. By contrast, in this study, Illumina sequencing of ammonia monooxygenase gene identified OTUs closely associated to different AOB species (Figure 1), confirming, AOB are inhabiting seeds.

Although our study used only *E. nutans* plants, the presence of AOB in seeds collected from all four locations of the QTP suggests a widespread phenomenon. Testing the presence of AOB in seeds of other plant species in different ecoregions is an important extension of this research. Plant type has been previously identified as an important factor determining the seed bacterial community (Wassermann et al., 2019; Liang et al., 2021), and hence the presence of different AOB community compositions in seeds of different plant species is highly likely. Indeed, we found that the AOB community composition in seeds was geographically varied (Figure 1A and Supplementary Table 2), indicating an environmental contribution toward plant selection of AOB in seeds. This observation is consistent with the findings of Morales Moreira et al. (2021), who concluded that the field environmental condition was one of the critical factors contributing to the seed-associated microbial assemblage in wheat, canola, and lentil seeds. The AOB survey of seeds collected from the four locations in the present study identified AOB belonging to the genera *Nitrosospira, Nitrosovibrio*, and *Nitrosomonas* (Figure 1) and represented known AOB clusters identified by previous phylogenetic studies (Boyle-Yarwood et al., 2008; Chen et al., 2011). Most sequences associated with AOB strains in seeds across the four locations belonged to *Nitrosospira* cluster 3 (Figure 1A and Supplementary Table 2), which is an AOB lineage ubiquitous across soils (Fierer et al., 2009), perhaps indicating a link between seed and soil AOB. *Nitrosospira multiformis* was the most dominant AOB strain in seeds across the four locations, ranging from 46.4 to 65.2% (average: 58.5 ± 8.3%). Comparing currently available complete genome sequencing of AOB, Norton et al. (2008) commented that *Nitrosospira multiformis* is an organism that can adapt to diverse environmental conditions as they have the highest abundance of signal transducer systems. Such physiological capabilities may have contributed to their dominance in seeds. The AOB survey in the different plant parts of *E. nutans* growing at site MQ also identified *Nitrosospira* cluster 3 to be dominant in seeds and *Nitrosospira multiformis* was the most dominant AOB strain in seeds, with a relative abundance of 74% (Supplementary Table 3).

The richness of the sequences associated with AOB strains was significantly greater in seeds compared to leaves, roots and soil (Figure 3A), and the AOB community composition in seeds (beta diversity) was dissimilar to that in leaves, roots and soil (Figure 3B). Thus, the indication is that seeds host a diverse pool of AOB for selective recruitment by plants and, possibly as a result, we found several sequences associated with AOB strains shared in seeds and other plant parts (Figure 2). A core AOB community with three sequences associated with AOB strains—*Nitrosospira* sp. Nsp17, *Nitrosospira* sp. PJA1 and *Nitrosovibrio* sp. RY3C—was found in all plant parts as well as in soil (Figure 2). Both seeds and soil alike hosted greater numbers of unique sequences associated with AOB strains (Figure 2) and also shared in leaves and roots, indicating both soil and seeds may have provided a pool of AOB for selective recruitment by plants. Rochefort et al. (2021) reported strong selection from seed and soil communities during microbiota assembly to *Brassica napus* genotypes, with 8–32% of soil taxa and 0.8–1.4% of seed-borne taxa colonizing seedlings.

Our experiments clearly demonstrated that seeds can be a source for associated AOB in plants. When seeds were germinated in a sterilized nutrient-rich plant growth medium, all sequences associated with AOB strains identified in seeds appeared in leaves, roots and root medium either at 12 or 39 days (Table 1). Our results are consistent with other studies that demonstrated seed-associated microbiota were transmitted to leaves and roots of seedlings (Quesada-Moraga et al., 2014; Abdelfattah et al., 2021; Rochefort et al., 2021). In 12-day-old plants, AOB were recovered only in roots and plant growth medium but not in leaves, suggesting AOB migration after seed germination may have been influenced by the NH_4_^+^ substrate availability. Another possibility for why AOB were not observed in leaves of 12-day-old plants but were detected in leaves of 39-day-old plants could be because the AOB population in leaves of plants was initially below sequencing detection levels, later proliferating 39 days after their transmission to the developing seedling.

Three sequences associated with AOB strains found in the seedlings were observed in the second-generation seeds of offspring plants, supporting the view of vertical transmission of seed microbiota (Tannenbaum et al., 2020; Abdelfattah et al., 2021). However, it was interesting to note that *Nitrosospira multiformis*—the most dominant AOB found in seeds in this study—was not vertically transmitted to next-generation seeds, despite its presence in 39-day-old plants. *Nitrosospira* sp. Nsp17—the second most dominant AOB found in seeds and also observed in 39-day-old plants—were vertically transmitted to next-generation seeds. The other two AOB vertically transmitted to next-generation seeds—*Nitrosospira briensis* and *Nitrosomonas nitrosa*—were not observed in 39-day-old plants. These results suggest that the AOB composition in plants can be transient in different plant stages, perhaps resulting in different compositions between the reproductive and vegetative stages. Microbial richness differences in progeny seeds have also been observed previously (Tannenbaum et al., 2020). Potential mechanisms that could be used by endophytic bacteria to reach the reproductive organs and eventually the seeds have been discussed by Frank et al. (2017). Understanding the critical factors that determine plant selection of AOB for the next generation will be an important area of future research.

Our observation of NO_3_^–^ production after seed germination and during seedling growth in plant growth medium, which was supplemented only with NH_4_^+^ as the N source, provided evidence that AOB transmitted from seeds to root medium contributed to the nitrification. It is interesting that we observed both NO_2_^–^ and NO_3_^–^ production after seed germination and seedling growth, indicating not only AOB but also NO_2_^–^ oxidizing bacteria (e.g., *Nitrobacter, Nitrospira*) were also transmitted from seeds to the plant growth medium. This result demonstrates the functional role of seed-inhabiting bacteria in the soil N cycle. Our experimental plant growth medium was sterile, but under natural conditions soils would harbor many different strains of AOB with varied rates and efficiency of nitrification. It would be interesting to test whether the seed-borne AOB would be transmitted to soil composed of a natural-soil AOB community and contribute to nitrification. Our observation of shared sequences associated with AOB strains in seeds and soil beneath *E. nutans* plants at site MQ suggests this is happening, but concrete evidence by tracking tagged AOB movements may be required to confirming this assertion.

Similarly, further studies are warranted to explore the benefits of allowing AOBs to be carried inside seeds and allowing them to live inside plant tissues in a symbiotic manner. We can speculate that plant AOB symbiotic associations may be related to plant preferential N source (NH_4_^+^ or NO_3_^–^) (Britto and Kronzucker, 2013), as well as the NH_4_^+^ toxicity tolerance of plants (Britto and Kronzucker, 2002). Plant and soil AOB exchange would allow plants to regulate their preferred N source in the rhizosphere by transmitting AOBs with varied efficiency to facilitate nitrification. Using eight plant species collected from three European grassland sites, Cantarel et al. (2015) showed that nitrification was strongly correlated to plant affinity for NH_4_^+^. The plants that were exposed to a high degree of NH_3_ and NH_4_^+^ uptake from soil can experience NH_4_^+^ toxicity, but some plant species have adopted different mechanisms to alleviate NH_4_^+^ toxicity (Britto and Kronzucker, 2002). Use of plant associated AOBs for detoxifying NH_4_^+^ could be one such mechanism that has not been explored before. For example, Papen et al. (2002) demonstrated that *in situ* uptake of NH_3_ by AOB residing inside spruce needles exposed to a high level of atmospheric NH_3_ perhaps prevented NH_3_ toxicity while at the same time supplying N to spruce trees.

In agricultural systems, N management is heavily focused on N transformations occurring in soil, but our recent studies on the influence of plants on the N cycle (Bowatte et al., 2014, 2015, 2016; Li et al., 2022), as well as the results of the present study, suggest that the extent of the involvement of plants in the N cycle is far greater than previously thought. Our findings are important for current efforts in minimizing N losses from agricultural systems, with significant interest having recently emerged regarding how plants influence N transformations in soil (Moreau et al., 2019), but especially as part of efforts looking to find methods that promote the minimization of environmental pollution caused by NO_3_^–^ leaching and nitrous oxide emissions. Most studies have focused on soil nitrification, as reducing nitrification in agricultural soils is often considered desirable for improving N use efficiency (Coskun et al., 2017). Plants can impact soil nitrification by adopting various N acquisition strategies (Abalos et al., 2018), releasing biological nitrification inhibitors (Subbarao et al., 2009), and supporting a unique nitrifying community (Ma et al., 2020). A fascinating question arising from our study is whether seed-borne AOB that migrate to seedlings and the root zone would play a critical role in establishing a keystone community leading to a unique AOB community in the plants and rhizosphere. Understanding the ways that microbes associated with seed contribute to the future plant microbiome is an emerging area of research as optimizing the seed microbiome of agricultural crops could bring huge benefits to plant breeding and crop improvement (Adam et al., 2016; Gopal and Gupta, 2016; Berg and Raaijmakers, 2018). For example, Mitter et al. (2017) discovered that specific beneficial endophytic bacterium introduced to the flowers of parent plants can be transferred to the seed microbiome and then passed on into offspring generations for the express beneficial growth traits. This study provided evidence for seed microbes that can directly influence the subsequent environmental performance of the plant, providing an important opportunity for plant improvement that also benefits environmental protection.

## Conclusion

We present evidence in this study: (a) endophytic AOB in *E. nutans* seeds; (b) transmission of AOB in seeds to offspring plants; (c) vertical transmission of endophytic AOB; and (d) a seed borne AOB contribution to nitrification. We believe the observation that AOB can be seed endophytes and their potential contribution to soil nitrification is important as it can be a first step in developing future plant-based manipulations of the soil N cycle. Future studies should explore whether seed borne AOB that migrate to seedlings and the root zone would play a critical role in establishing a keystone community leading to a unique AOB community in the plants and rhizosphere. If this is the case, then plants may have the potential to “pass on” their characteristic AOB from generation to generation, providing an important opportunity for plant improvement for environmental protection.

## Data availability statement

The original contributions presented in this study are included in the article/Supplementary material, further inquiries can be directed to the corresponding author.

## Author contributions

SB conceived the idea. SB and DL designed the research and wrote the manuscript. DL performed the experiments and analyzed the data. Both authors contributed to the article and approved the submitted version.
