# Supplementary material for: Seed endophytic ammonia oxidizing bacteria in Elymus nutans transmit to offspring plants and contribute to nitrification in the root zone
Source: Front Microbiol. 2022 Nov 29;13:1036897. doi: 10.3389/fmicb.2022.1036897 (PMC9744808; doi:10.3389/fmicb.2022.1036897)
Supplement: Supplementary file 1 [file Data_Sheet_1.docx]

**Supplementary Table 1:** The geographic and climate information of four locations of *Elymus nutans* seed collection in Qinghai Tibetan Plateau

| Sampling site | Longitude | Latitude | Elevation (m) | The annual average temperature (℃) | The annual average precipitation (mm) |
| --- | --- | --- | --- | --- | --- |
|  |  |  |  |  |  |
| Hongyuan (HY) | 102.33'07'' | 31°47'34'' | 3500 | 1.1 | 738 |
| Maqu (MQ) | 102°00' | 33°59' | 3529 | 1.2 | 616 |
| Luqu (LQ) | 102°34'4'' | 34°24'6'' | 3471 | 1.2 | 610 |
| Guoluo (GL) | 100°39'3'' | 34°20'3'' | 3933 | -4 | 760 |

**Supplementary Table 2:** The relative abundance of OTUs associated with different AOB strains in *Elymus nutans* seeds collected from four locations. The values are mean ± standard deviation. The uppercase letters indicate the significance of differences between four geographical locations, and lowercase letters indicate the significance of differences between taxa, significance was calculated by Dunn-Sidak test (p<0.05)

| Ammonia oxidizing bacteria | Ma qu | Lu qu | Hong yuan | Guo luo |
| --- | --- | --- | --- | --- |
| *Nitrosomonas eutropha* | 0.549±0.008 c | - | - | - |
| *Nitrosomonas nitrosa* | 0.171±0.002 Ac | 0.522±0.009 Ad | 0.004±0.001Ad | - |
| *Nitrosomonas sp.* | 0.541±0.009 Ac | - | 0.167±0.003 Ad | 0.525±0.009 d |
| *Nitrosomonas sp. IWT514* | - | - | - | - |
| *Nitrosomonas sp. Nm41* | 0.417±0.007 c | - | - | - |
| *Nitrosomonas sp. Nm47* | 0.217±0.003 c | - | - | - |
| *Nitrosomonas sp. Nm58* | 0.138±0.002 c | - | - | 0.070±0.001 Ad |
| *Nitrosospira briensis* | 0.027±0.001 Ac | - | - | 59.837±0.013 ABa |
| *Nitrosospira multiformis* | 46.439±0.166 Aa | 62.479±0.052 ABa | 65.222±0.071 Ba | - |
| *Nitrosospira sp. cT2F* | - | 0.363±0.006 d | - | 0.374±0.006 Ad |
| *Nitrosospira sp. 9SS1* | 0.612±0.003 Ac | 0.047±0.001 Ad | 0.027±0.001 Ad | 0.440±0.003 Bd |
| *Nitrosospira sp. En13* | 24.209±0.232 Ab | 0.555±0.005 Bd | 0.409±0.007 Bd | - |
| *Nitrosospira sp. EnI299* | 0.005±0.001 Ac | - | 0.018±0.001 Ad | - |
| *Nitrosospira sp. III7* | 0.032±0.001 c | - | - | 0.201±0.002 d |
| *Nitrosospira sp. KAN8* | 0.129±0.001 c | 0.551±0.008 d | 0.062±0.001 d | 21.563±0.053 Ab |
| *Nitrosospira sp. L115* | 12.839±0.057 Abc | 19.286±0.047 Ab | 20.546±0.072 Ab | - |
| *Nitrosospira sp. LT1FMf* | - | - | 0.009±0.001 d | - |
| *Nitrosospira sp. LT2MFa* | - | - | 0.413±0.007 d | - |
| *Nitrosospira sp. Np39-19* | - | - | 0.189±0.003 d | 3.938±0.018 Acd |
| *Nitrosospira sp. Nsp17* | 3.642±0.008 Ac | 2.524±0.027 Ad | 0.787±0.014 Acd | - |
| *Nitrosospira sp. Nsp40* | 0.009±0.001 c | - | - | - |
| *Nitrosospira sp. Nsp58* | 0.008±0.001 Ac | - | 0.026±0.001 Ad | 3.768±0.008 Acd |
| *Nitrosospira sp. PJA1* | 1.91±0.008 Ac | 2.787±0.017 Ad | 1.870±0.017 Acd | 6.844±0.031 Ac |
| *Nitrosospira sp. TcH711* | 6.145±0.022 Abc | 8.074±0.016 Ac | 7.809±0.044 Ac | - |
| *Nitrosovibrio sp. FJI82* | 0.159±0.003 c | - | - | 2.434±0.017 Acd |
| *Nitrosovibrio sp. RY3C* | 1.802±0.003 Ac | 2.808±0.009 Ad | 2.442±0.014 Acd | 0.004±0.001 Ad |
| *Nitrosovibrio tenuis* | - | 0.004±0.001 Ad | - | - |

**Supplementary Table 3:** The relative abundance of OTUs associated with different AOB strains in different plant parts and soils of *Elymus nutans* growing in alpine meadows at Maqu (MQ)

| Seed | Relative abundance | Leaf | Relative abundance | Root | Relative abundance | Soil | Relative abundance |
| --- | --- | --- | --- | --- | --- | --- | --- |
| *Nitrosospira multiformis* | 74.06% | *Nitrosospira sp. Nsp17* | 33.66% | *Nitrosospira sp. Nsp40* | 32.64% | *Nitrosospira briensis* | 28.89% |
| *Nitrosospira sp. Nsp17* | 10.17% | *Nitrosospira sp. Np39-19* | 33.55% | *Nitrosospira sp. PJA1* | 31.39% | *Nitrosospira sp. Nsp17* | 22.72% |
| *Nitrosospira sp. PJA1* | 6.98% | *Nitrosospira multiformis* | 16.72% | *Nitrosospira sp. III7* | 17.75% | *Nitrosospira sp. Ka4* | 17.73% |
| *Nitrosospira sp. L115* | 4.01% | *Nitrosovibrio sp. RY3C* | 8.52% | *Nitrosospira sp. Nsp17* | 15.55% | *Nitrosospira sp. Np39-19* | 12.39% |
| *Nitrosospira sp. III7* | 2.17% | *Nitrosospira sp. L115* | 5.07% | *Nitrosospira briensis* | 1.75% | *Nitrosospira sp. CT2F* | 8.56% |
| *Nitrosospira sp. 24C* | 0.83% | *Nitrosospira sp. Nsp40* | 1.19% | *Nitrosospira sp. Np39-19* | 0.70% | *Nitrosospira sp. Wyke2* | 3.82% |
| *Nitrosovibrio sp. FJI82* | 0.48% | *Nitrosospira sp. EnI299* | 0.76% | *Nitrosospira multiformis* | 0.10% | *Nitrosospira sp. Nsp5* | 3.20% |
| *Nitrosospira sp. 9SS1* | 0.42% | *Nitrosospira sp. PJA1* | 0.54% | *Nitrosovibrio sp. RY3C* | 0.10% | *Nitrosospira sp. Nsp40* | 0.94% |
| *Nitrosospira sp. Wyke8* | 0.25% |  |  | *Nitrosospira sp.* | 0.02% | *Nitrosospira sp. 40KI* | 0.71% |
| *Nitrosospira sp. En13* | 0.23% |  |  | *Nitrosospira sp. TCH711* | 0.00% | *Nitrosovibrio sp. RY3C* | 0.60% |
| *Nitrosospira sp. AF* | 0.17% |  |  |  |  | *Nitrosospira sp. PJA1* | 0.26% |
| *Nitrosomonas nitrosa* | 0.14% |  |  |  |  | *Nitrosospira sp. 9SS1* | 0.09% |
| *Nitrosospira sp. TCH711* | 0.03% |  |  |  |  | *Nitrosospira sp. III2* | 0.07% |
| *Nitrosospira briensis* | 0.02% |  |  |  |  | *Nitrosospira sp. Nsp12* | 0.04% |
| *Nitrosospira sp.* | 0.01% |  |  |  |  |  |  |
| *Nitrosovibrio sp. RY3C* | 0.01% |  |  |  |  |  |  |

**Supplementary Table 4:** The relative abundance of OTUs associated with different AOB strains transmitted to leaf, root, and root medium of offspring *Elymus nutans* seedlings at 12 and 39 days.

| Day 12 | | | | Day 39 | | | | | |
| --- | --- | --- | --- | --- | --- | --- | --- | --- | --- |
| Root | Relative abundance | Root medium | Relative abundance | Leaf | Relative abundance | Root | Relative abundance | Root medium | Relative abundance |
| *Nitrosospira sp. III7* | 46.23% | *Nitrosospira sp. III7* | 53.39% | *Nitrosospira sp. Np39-19* | 35.12% | *Nitrosospira sp. PJA1* | 54.81% | *Nitrosospira sp. PJA1* | 31.94% |
| *Nitrosospira briensis* | 23.36% | *Nitrosospira multiformis* | 30.79% | *Nitrosospira sp. III7* | 33.13% | *Nitrosovibrio sp. RY3C* | 28.18% | *Nitrosovibrio sp. RY3C* | 26.67% |
| *Nitrosospira sp. Wyke2* | 14.97% | *Nitrosospira sp. Nsp57* | 13.84% | *Nitrosospira multiformis* | 31.63% | *Nitrosospira sp. Np39-19* | 11.12% | *Nitrosospira sp. Np39-19* | 11.68% |
| *Nitrosospira sp. Nsp17* | 3.08% | *Nitrosospira sp. Nsp12* | 0.51% | *Nitrosospira sp. PJA1* | 0.06% | *Nitrosospira sp. Nsp40* | 1.96% | *Nitrosospira sp. Nsp17* | 11.64% |
| *Nitrosospira multiformis* | 2.78% | *Nitrosospira sp. PJA1* | 0.51% | *Nitrosospira sp. B6* | 0.04% | *Nitrosospira sp. Nsp17* | 1.96% | *Nitrosospira multiformis* | 10.35% |
| *Nitrosospira sp. Np39-19* | 2.41% | *Nitrosospira sp. TCH711* | 0.34% | *Nitrosovibrio sp. RY3C* | 0.01% | *Nitrosospira sp. EnI299* | 1.96% | *Nitrosospira sp. Nsp40* | 7.44% |
| *Nitrosospira sp. Nsp12* | 1.69% | *Nitrosomonas nitrosa* | 0.17% | *Nitrosospira sp. Nsp17* | 0.01% |  |  | *Nitrosospira sp. Nl5* | 0.27% |
| *Nitrosospira sp. PJA1* | 1.66% | *Nitrosospira sp. Wyke2* | 0.17% |  |  |  |  |  |  |
| *Nitrosospira sp. Nsp40* | 1.27% | *Nitrosospira sp. Nsp17* | 0.17% |  |  |  |  |  |  |
| *Nitrosospira sp. Nsp5* | 0.64% | *Nitrosospira sp.* | 0.11% |  |  |  |  |  |  |
| *Nitrosospira sp.* | 0.40% | *Nitrosospira sp. III2* | 0.00% |  |  |  |  |  |  |
| *Nitrosospira sp. III2* | 0.35% |  |  |  |  |  |  |  |  |
| *Nitrosospira sp. TCH711* | 0.33% |  |  |  |  |  |  |  |  |
| *Nitrosovibrio sp. RY3C* | 0.30% |  |  |  |  |  |  |  |  |
| *Nitrosospira sp. Ka4* | 0.20% |  |  |  |  |  |  |  |  |
| *Nitrosospira sp. Nsp57* | 0.11% |  |  |  |  |  |  |  |  |
| *Nitrosospira sp. CT2F* | 0.10% |  |  |  |  |  |  |  |  |
| *Nitrosomonas sp. LT-4* | 0.03% |  |  |  |  |  |  |  |  |
| *Nitrosospira sp. Wyke8* | 0.03% |  |  |  |  |  |  |  |  |
| *Nitrosospira sp. 40KI* | 0.03% |  |  |  |  |  |  |  |  |
| *Nitrosospira sp. Ka3* | 0.03% |  |  |  |  |  |  |  |  |
| *Nitrosomonas nitrosa* | 0.01% |  |  |  |  |  |  |  |  |

**Supplementary Table 5**: The relative abundance of OTUs associated with different AOB strains retrieved from second generation seeds of *Elymus nutans* plants grown for 15 months in greenhouse.

| Ammonia oxidizing bacteria | Relative abundance |
| --- | --- |
| *Nitrosospira briensis* | 67.834% |
| *Nitrosomonas nitrosa* | 31.502% |
| *Nitrosospira sp. Nsp17* | 0.665% |
