# Supplementary material for: Seed endophytic ammonia oxidizing bacteria in Elymus nutans transmit to offspring plants and contribute to nitrification in the root zone
Source: Front Microbiol. 2022 Nov 29;13:1036897. doi: 10.3389/fmicb.2022.1036897 (PMC9744808; doi:10.3389/fmicb.2022.1036897)
Supplement: Supplementary file 2 [file Table_2.DOCX]

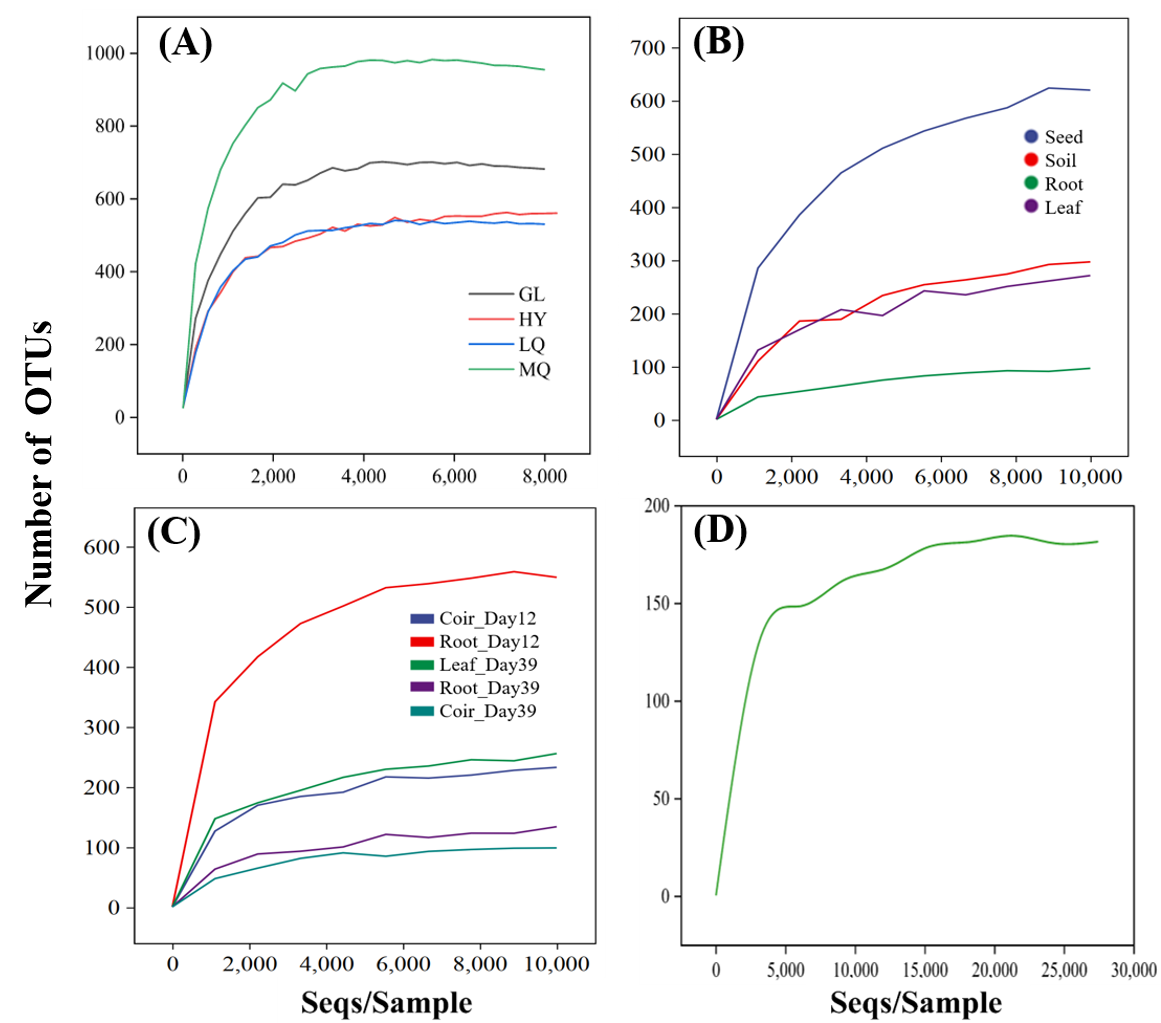


**Supplementary Figure 1** Rarefaction curves for the observed ammonia oxidizing bacteria OTUs in *Elymus nutans* seeds collected from four locations (**A**), different parts of *Elymus nutans* plants growing in alpine meadows at Maqu (**B**), leaves, roots and coir medium at days 12 and 39 during greenhouse experiment (**C**) and the second generation of seeds (**D**) (n=3).
